# Gene Expression Profiling of Breast Cancer Brain Metastasis

**DOI:** 10.1038/srep28623

**Published:** 2016-06-24

**Authors:** Ji Yun Lee, Kyunghee Park, Eunjin Lee, TaeJin Ahn, Hae Hyun Jung, Sung Hee Lim, Mineui Hong, In-Gu Do, Eun Yoon Cho, Duk-Hwan Kim, Ji-Yeon Kim, Jin Seok Ahn, Young-Hyuck Im, Yeon Hee Park

**Affiliations:** 1 Division of Hematology-Oncology, Department of Medicine, Samsung Medical Center, Sungkyunkwan University School of Medicine, Seoul, Korea; 2 Samsung Genomic Institute, Samsung Biological Research Institute, Samsung Medical Center, Sungkyunkwan University School of Medicine, Seoul, Korea; 3 Department of Health Sciences and Technology, SAIHST, Sungkyunkwan University School of Medicine, Seoul, Korea; 4 Center of Companion Diagnostics, Innovative Cancer Medicine Institute, Samsung Medical Center, Sungkyunkwan University School of Medicine, Seoul, Korea; 5 Department of Molecular Cell Biology, Samsung Biomedical Research Institute, Sungkyunkwan University School of Medicine, Suwon, Korea

## Abstract

The biology of breast cancer brain metastasis (BCBM) is poorly understood. We aimed to explore genes that are implicated in the process of brain metastasis of primary breast cancer (BC). NanoString nCounter Analysis covering 252 target genes was used for comparison of gene expression levels between 20 primary BCs that relapsed to brain and 41 BCBM samples. PAM50-based intrinsic subtypes such as HER2-enriched and basal-like were clearly over-represented in BCBM. A panel of 22 genes was found to be significantly differentially expressed between primary BC and BCBM. Five of these genes, *CXCL12*, *MMP2*, *MMP11*, *VCAM1*, and *MME*, which have previously been associated with tumor progression, angiogenesis, and metastasis, clearly discriminated between primary BC and BCBM. Notably, the five genes were significantly upregulated in primary BC compared to BCBM. Conversely, *SOX2* and *OLIG2* genes were upregulated in BCBM. These genes may participate in metastatic colonization but not in primary tumor development. Among patient-matched paired samples (*n* = 17), a PAM50 molecular subtype conversion was observed in eight cases (47.1%), with a trend toward unfavorable subtypes in patients with the distinct gene expression. Our findings, although not conclusive, reveal differentially expressed genes that might mediate the brain metastasis process.

Brain metastasis (BM) remains an intractable clinical problem despite notable advances in the treatment of breast cancer (BC). The prevalence of breast cancer brain metastasis (BCBM) has been reported to range from 10–30%^1^,^2^. Therapeutic approaches for the management of metastatic brain lesions are mostly local and palliative, such as surgical resection, sterotactic radiosurgery (SRS), and/or whole-brain radiation therapy (WBRT), and result in a median survival of 6–18 months^3^,^4^,^5^. The poor prognosis is mainly because systemic treatments with efficacy in the brain microenvironment are limited.

Many genes showing increased expression that correlates with brain metastasis have been identified, and some have been shown to play a causal role in this process^6^. Gene expression analysis between brain metastatic and parental breast cancer cell lines performed by Bos *et al*. indicated that *HBEGF, COX2*, and *ST6GALNAC5* mediate brain metastasis^7^. Zhang *et al*. identified a potential signature of BCBM in human circulating tumor cells overexpressing HER2/EGFR/HPSE/Notch1^8^. Bollig-Fischer *et al*. reported copy number gains of *SOX1*, *PIK3CA*, *NTRK1*, *GNAS*, *CTNNB1*, and *FGFR1* in BCBM tissues^9^. Matched pair analysis of targeted sequencing data between primary BC and BCBM demonstrated that known drivers of primary breast cancer were frequently mutated in BCBM, including *TP53*, *MLH1*, *PIK3CA*, and *KIT*^10^. However, despite advances in our knowledge of the genetic basis for cancer metastasis, comprehensive genomic characterization of BCBM for development of biomarkers and molecularly targeted therapies remains an unmet need.

Recent genome-wide searches for metastasis-associated events have focused more on gene expression changes than on mutations or gene copy-number alterations^11^. The NanoString nCounter Dx Analysis System has been shown to provide more precise and accurate measures of mRNA expression levels in formalin-fixed, paraffin-embedded (FFPE) tissue than polymerase chain reaction (PCR)^12^. By analyzing expression arrays with NanoString nCounter in surgically resected BCBM and primary BC relapsing to brain, we aimed to investigate molecules associated with the brain metastasis processes.

## Results

### Patient characteristics

A total of 44 patients with brain metastasis from breast cancer were included in this study (Supplementary Figure 1). Patient demographics are summarized in Table 1. Median age at diagnosis of BCBM was 48 years. The majority of patients were premenopausal woman (79.5%) and the most common histology was invasive ductal carcinoma (88.1%). Five (11.9%) patients were initially diagnosed with stage IV metastatic disease. Among 44 patients, 19 (43.2%) had distant metastasis prior to the formation of brain lesions. The most common sites of metastasis prior to BCBM were lung, bone, and liver. Median survival from diagnosis of BCBM was 23.2 months (range, 3.1–79.8 months). We observed a difference in overall survival from time of initial diagnosis of BC according to the subtype of BCBM (Supplementary Figure 2A). However, the subtype of BCBM did not influence survival from time of BCBM (Supplementary Figure 2B).

### Distribution pattern of the subtypes

**Figure 1:**
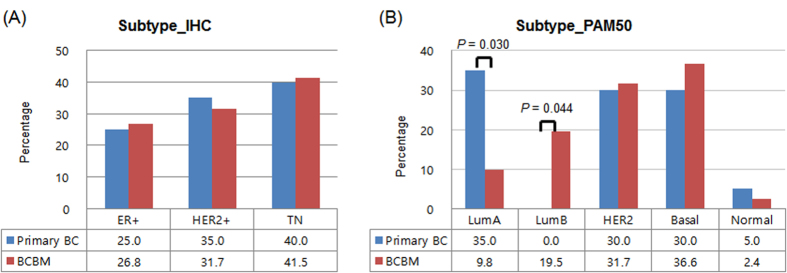
Frequency of subtypes according to immunohistochemistry (**A**) and PAM50 by NanoString nCounter assay (**B**).

The overall subtype distribution based on immunohistochemical (IHC) and PAM50 is shown in Fig. 1 and Supplementary Table 1. When we compared the IHC subtypes between primary BC (*n* = 20) and BCBM (*n* = 41), a similar pattern was seen in the two cohorts (*P* = 0.934): triple negative BC (TNBC) and HER2+ irrespective of hormone receptor status was the most common subtypes in both cohorts (Fig. 1A). In contrast, a significant difference in the distribution pattern of PAM50 subtypes was identified between primary BC and BCBM (Fig. 1B). Among the primary BC cohort, the distribution by PAM50 subtype included 35.0% luminal A, 30.0% HER2-enriched, 30.0% basal-like, and 5.0% normal-like. The distribution of PAM50-based subtype in the BCBM cohort was estimated to be 36.6% basal-like, 31.7% HER2-enriched, 19.5% luminal B, 9.8% luminal A, and 2.4% normal-like. Luminal A type was more frequent in primary BC than in BCBM (*P* = 0.030) whereas the luminal B type was only observed in the BCBM cohort (*P* = 0.044) (Fig. 1B).

### Identification of genes that are differentially expressed between primary BC and BCBM

**Figure 2:**
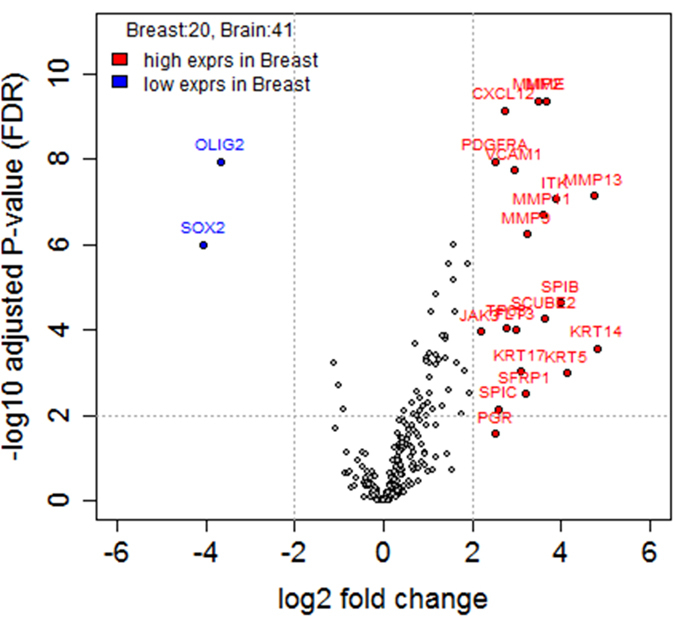
Gene expression profiles of the 20 primary breast cancers (BC) compared to those of the 41 breast cancer brain metastases (BCBM). Volcano plots show the distribution of the fold changes in gene expression. Genes with absolute fold change ≥2 and adjusted *P*-value *FDR* < 0.05 are indicated in red (high expression in primary BC compared to BCBM) and blue (low expression in primary BC compared to BCBM). Comparisons were analyzed using Student’s t-test.

To identify patterns of gene expression associated with BM, we performed a NanoString expression assay of 252 target genes and five reference genes using mRNA extracted from FFPE samples (Supplementary Table 2). The gene list obtained from a class comparison between primary BC and BCBM was filtered based on the criteria of a fold change ≥2 and a false discovery rate (FDR) < 0.05 (Supplementary Figure 3). As a result, 20 upregulated genes and two downregulated genes were identified in primary BC (Fig. 2 and Supplementary Table 3). The genes that were upregulated in primary BC included *MMPs* (*MMP2*, *MMP9*, *MMP11*, *MMP13*, and *MME*), *KRTs* (*KRT5*, *KRT14*, and *KRT 17*), *VCAM1*, *CXCL12*, *SCUBE2*, *TP63*, and *SFRP*. Expression levels of *SOX2* and *OLIG2* were downregulated in primary BC compared to BCBM.

**Figure 3:**
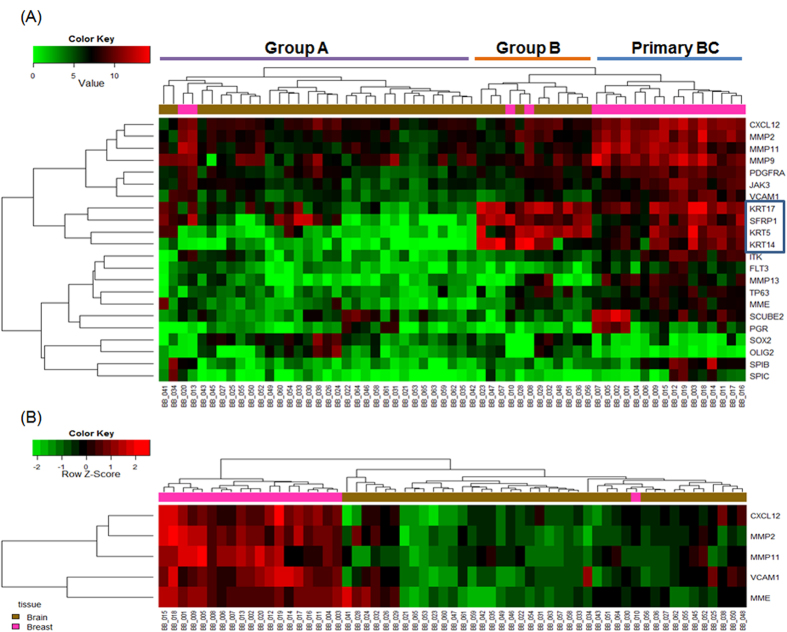
Heat map showing differences in the expression patterns of 22 genes with absolute fold change ≥2 and *FDR* < 0.05 (**A**) and 5 genes after pathway activity inference using condition-responsive genes analysis (**B**). Hierarchical clustering was performed with the complete linkage method using the Euclidean distance measure. Comparisons were analyzed using Student’s t-test.

We performed hierarchical clustering analysis of the 22 identified differentially expressed transcripts to visualize the gene expression profiles of primary BC and BCBM (Fig. 3A). Two distinct clusters were evident in the BCBM group: group A, which was clearly separated from primary BC, and group B, which was similar to primary BC. BCBM samples that closely resembled primary BC were mainly TN type by IHC and basal-like subtype by PAM50 (Supplementary Table 4). Genes that were overexpressed in both primary BC and BCBM that was similar to primary BC included *KRT5*, *KRT14*, *KRT17*, and *SFRP1*.

Next, we conducted pathway activity inference using condition-responsive genes (PAC)^13^ analysis to identify gene sets among the 22 genes that optimize the discriminative power. By PAC analysis, five genes (*CXCL12*, *MMP2*, *MMP11*, *VCAM1*, and *MME*) were identified as the best classifiers for discriminating primary BC and BCBM (Fig. 3B). These five genes were highly upregulated in primary BC compared to BCBM.

### Gene expression patterns in patient-matched paired samples

Next, we explored patient-matched paired samples of primary BC and BCBM and observed discordant expression of PAM50 molecular subtypes and IHC subtypes between primary BC and BCBM (Table 2). PAM50 molecular subtype conversion was observed in 8/17 (47.1%) matched pairs. Among six luminal A types in primary BC, a molecular subtype change was observed in five cases: three to HER2-enriched and two to luminal B subtype. By IHC, two cases had discordant ER expression between primary BC and BCBM, both involving loss of ER.

**Figure 4:**
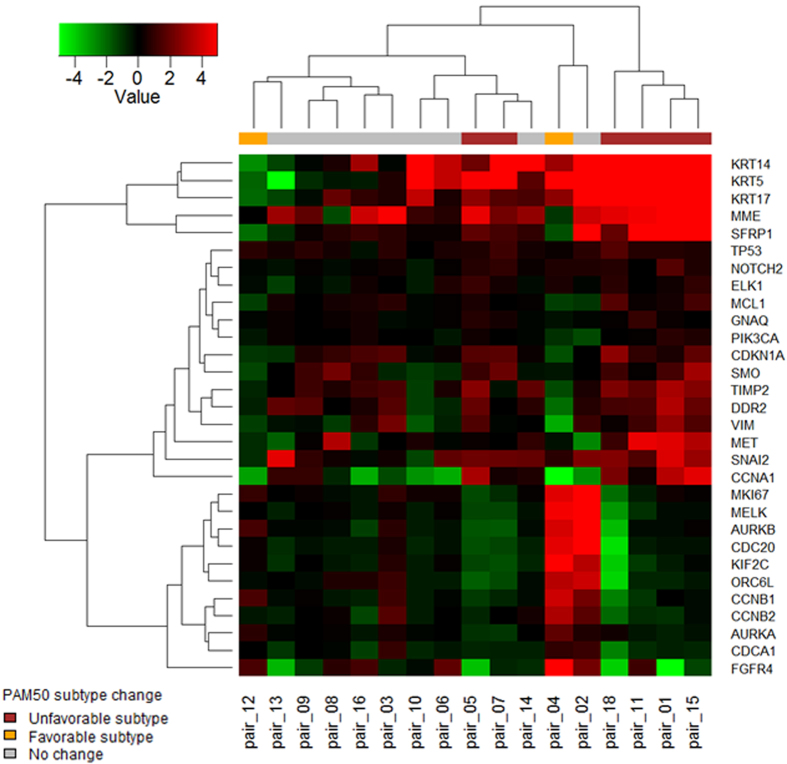
Heat map showing the top 30 significant genes that were differentially expressed between primary BC and BCBM in patient-matched paired samples. Red, pairs that converted toward the unfavorable subtype; orange, pairs that converted toward the favorable subtype; gray, pairs that did not change subtype. Hierarchical clustering was performed with the complete linkage method using the Euclidean distance measure.

To further examine the genes indicated in BM, gene expression analysis was performed on patient-matched paired samples (*n* = 17) (Supplementary Figure 4). Figure 4 shows the top 30 significant genes that were differentially expressed between matched primary BC and BCBM samples from individual patients. Interestingly, hierarchical clustering analysis revealed that the group that underwent conversion to unfavorable subtypes during metastasis included genes that were upregulated in primary BC compared with BCBM such as *KRT14*, *KRT5*, *KRT17*, *MME*, and *SFRP1*, and genes that were downregulated, including *MKI67*, *AURKB*, *CDC20*, and *KIF2C*.

### Contribution of p53 mutation to metastasis

Our previous study showed that *TP53* mutation was the most common mutation in primary BC (38.9%) and BCBM (59.5%)^10^. Compared to the overall frequency of p53 mutation in BC (~20%)^14^, p53 mutations were highly over-represented in our cases of primary BC and BCBM. For identification of differentially expressed genes (DEGs) that are influenced by p53 mutation, we compared the gene expression signature between wild-type and mutant p53 groups (Supplementary Figure 5). Notably, significant downregulation of *MAPT* for the total cohort and *ERBB4* for the BCBM cohort was observed in the mutant p53 group using criteria of a fold change ≥2 and *P* value < 0.01 (Supplementary Figure 6). In addition, expression of *CDKN1A* was decreased in the mutant p53 group, although this was not statistically significant.

## Discussion

The frequency of diagnosis of BCBM seems to be increasing as a result of improved imaging modalities and longer survival due to effective systemic control of the primary BC. Despite recent advances in molecular profiling associated with BM, the underlying biology remains unclear^15^,^16^,^17^. In this study, gene expression analysis by NanoString nCounter assay provided many candidate genes that may be associated with the BM process.

A higher incidence of BM has been correlated with BC molecular subtypes such as HER2 and TN types^18^,^19^. In the current study, HER2+ and TN subtypes accounted for 31.7% and 41.5% of cases in the BCBM cohort respectively. Compared to the proportion of BC patients categorized as HER2+ (12–22%) and TN (6–28%) in the literature^20^, these subtypes were clearly over-represented in BCBM. The distribution of PAM50-based intrinsic subtypes in the BCBM cohort was predominantly HER2-enriched (31.7%) or basal-like (36.6%) type. Based on this observation, we speculate that metastatic invasion into the brain may be the result of clonal selection favoring HER2+ or basal-like cell clones. In addition, with advances in treatments for BC that control systemic metastatic diseases at other organs, such as the monoclonal antibody trastuzumab, new challenges of controlling BCBM have emerged in cases of HER2+ BC^21^,^22^.

We identified 22 genes that were differentially expressed between primary BC and BCBM. Using hierarchical clustering analysis of these genes, BCBM samples were divided into two groups based on whether the gene expression signatures were different from or similar to those of primary BC. A five-gene expression signature including *CXCL12*, *MMP2*, *MMP11*, *VCAM1*, and *MME* clearly discriminated between primary BC and BCBM. Notably, these genes have been shown to be involved in processes necessary for metastasis; for example, genes associated with increased cancer cell growth, migration, adhesion, invasion, and regulation of angiogenesis were significantly highly expressed in primary BC compared to BCBM^23^,^24^,^25^,^26^,^27^,^28^,^29^. MMPs have long been associated with cancer cell invasion and metastasis through their activity in cleaving a diverse group of substrates including structural components of the extracellular matrix, growth-factor-binding proteins, receptor tyrosine kinases, cell-adhesion molecules, and other proteinases^23^,^24^. CXCL12-CXCR4 signaling promotes tumor growth and metastasis in BC by chemotaxis, proliferation of CXCR4+ cancer cells, and stimulation of angiogenesis^25^,^26^. Kang *et al*. reported that high CXCL12 levels correlate with increased metastasis and local recurrence in BC^27^. In this study, a positive correlation (correlation coefficient of 0.52) was shown between CXCL12 and CXCR4 (Supplementary Figure 7) Recent studies have shown that VCAM1 is aberrantly expressed in breast cancer cells and mediates prometastatic tumor-stromal interactions that are unique to lung and bone microenvironments^28^,^29^. Taken together, these findings suggest that upregulation of these genes in primary BC may be associated with a role in metastasis initiation and progression. Conversely, downregulation of these genes in BCBM indicates a need for cell growth rather than tumor cell motility or invasion. This suggests that the genomic instability of primary BC allows the acquisition of properties favoring the metastatic process^11^. In addition, the distant metastatic site is a largely nonpermissive environment, which means only a few cancer cells become metastatic^30^.

Four genes, *KRT5*, *KRT14*, *KRT17*, and *SFRP1*, were highly overexpressed in both primary BC and the subset of BCBM that was similar to BC. These findings suggest that these genes might be associated with predetermined traits for brain metastasis. According to Perou *et al*.^31^ and Solie *et al*.^32^, basal-like BCs that group together exhibit high expression of KRT5 and KRT17 and are associated with aggressive characteristics including relapse and reduced survival^33^. SFRP1 has been suggested to be a tumor suppressor through inhibition of Wnt/ß-catenin signaling^34^; however, emerging evidence has shown that these genes may also promote tumor growth^35^,^36^. For example, Qu *et al*. showed that SFRP1 is overexpressed in some gastric cancers and regulates cell growth and migration/invasion^36^, and is highly expressed in basal-like breast cancer and in brain relapses^37^. These contradictory findings can be explained by the genomic complexity of cancers and the underlying molecular mechanisms require further clarification.

The remarkable adaptation of tumor cells observed in metastasis is indicative of co-evolution occurring at specific metastatic organ microenvironments^38^,^39^. The brain presents a unique and complex tissue microenvironment and the colonization and formation of BCBM depends on interactions between the microenvironment and the colonizing metastatic breast cancer cells^40^. Surprisingly, we found that *SOX2* and *OLIG2* mRNA expression was increased in BCBM compared with the primary BC. SOX2 is one of the key transcriptional factors that control the unique properties of stem cells, especially in development of the central nervous system (CNS)^41^,^42^. Bolling-Fischer *et al*. identified stem cell pluripotency pathway enrichment including *SOX2* in BCBM specimens^9^. *OLIG2* encodes a basic helix-loop-helix (bHLH) transcription factor that is expressed in both the developing and mature CNS^43^. A recent study demonstrated that OLIG2 expression is restricted to neuroectodermally-derived tumors such as oligodendrogliomas and high-grade astrocytomas^44^. Park *et al*. showed that the brain microenvironment induces complete reprogramming of metastasized breast cancer cells, resulting in a gain of neuronal cell characteristics, which can also be induced by culture with astrocytes^45^. A recent study by Zhang *et al*. showed that PTEN loss in tumor cells promotes brain metastasis and that protein downregulation is epigenetically regulated by brain astrocytes^46^. *SOX2* and *OLIG2* seem to play important roles in phenotypic plasticity in brain microenvironments.

It is noteworthy that among the patient-matched paired samples, PAM50 molecular subtype conversion of BC was noted in 47.1% of cases. The most common molecular changes are conversion of luminal A into Her-2 enriched or luminal B subtypes that have aggressive clinical and biologic features^47^. When we compared the gene expression profiles in patient-matched paired samples, distinct differences in gene expression patterns between primary BC and BCBM were noted in the group that converted toward a high-risk subtype in BCBM. Expression of certain genes, such as *KRT14*, *KRT5, KRT17*, *MME*, and *SFRP1*, was highly upregulated and that of other genes, including *MKI67*, *AURKB*, *CDC20*, and *KIF2C*, was downregulated in primary BC compared with BCBM. Although the underlying mechanism of biologic conversion is unknown, these genes may play an important role in aggressiveness and the metastasis process.

According to a previous study by Lee *et al*., mutations in *TP53* were frequently observed in up to 60% of BCBMs^10^. The rate of *TP53* mutation varies among subtypes, with the highest frequency in basal-like (80%) and HER2-enriched (72%) subtypes and the lowest in luminal A (12%) and luminal B (29%) subtypes^48^. Indeed, the high frequency of *TP53* mutation in BCBM might be caused by an increase in basal-like and HER2-enriched subtypes of BCBM. p53 directly influences the transcription of genes involved in metastasis by binding to the promoters of a variety of genes related to cell motility, adhesion, and invasion^49^. Moreover, dysregulation of *TP53* target genes (i.e., lower expression of p53-activated genes and higher expression of p53-repressed genes) was significantly linked to the development of distant metastasis within 5 years of diagnosis^50^. In this study, *MAPT*, *ERBB4*, and *CDKN1A* were downregulated in the mutant p53 group compared with the wild-type p53 group. *CDKN1A* is a well-characterized p53 target gene with a confirmed p53 binding site in its promoter region^51^, whereas the role of *MAPT* and *ERBB4* in the metastasis processes as targets of p53 was not previously identified. Langerød *et al*. showed that the upregulated genes in carcinomas with a *TP53* mutation (e.g., *CCNB2*, *CDCA5*, and *CENPA*) were involved in the cell cycle and cell proliferation, whereas the downregulated genes (e.g., *IRS1*, *ESR1*, and *DNAL1*) were highly associated with ER status^52^. Further knowledge of the gene expression pattern of different *TP53* mutations is needed to understand their clinical relevance to p53-dependent metastasis.

Given that studies of the biology of BM have been limited by the lack of tissue availability, our analysis of DEGs between primary BC and BCBM represents a unique data set. However, there are several limitations. First, the relatively small sample size may provide an inaccurate representation of BCBM. Second, the set of 252 target genes was based on the PAM 50 gene set and previously defined gene signatures related to BC biology. The full analytical power cannot be achieved due to the insufficient number of genes. Third, there was no functional study to interrogate roles of the DEGs between primary BC and BCBM. To overcome this limitation we are currently planning to validate these genes and refine the preclinical models. Fourth, it is unclear whether these genes selectively mediate brain metastasis. To identify gene signatures linked specifically to BC metastasis to brain, an additional patient cohort with metastasis to other distant organs, but not to brain, is needed. Lastly, a better understanding of the role of tumor infiltrating immune cells in each step of the metastatic process will enable the development of new immunotherapeutic strategies to target these cells^53^.

Although our findings are not conclusive, we have identified DEGs between primary BC and BCBM that might mediate metastasis initiation and progression and provide a selective advantage in the brain microenvironment. Functional verification and clinical validation are needed to confirm candidate genes associated with BCBM.

## Methods

### Patient population

The study population consisted of patients with BC that had relapsed to the brain. Samples from 20 primary BCs and 41 BCBMs, including 17 patient-matched pairs, were collected after surgical resections performed at Samsung Medical Center. All patients provided written informed consent for the use of archival tissues with retrospective clinical data. This study was performed in accordance with the Declaration of Helsinki and approved by the Institutional Review Board of Samsung Medical Center (SMC 2013-12-155).

### Immunohistochemistry

Two experienced pathologists reviewed all pathology specimens to determine the following tumor characteristics: histologic and nuclear grades, primary tumor size, presence of lymphovascular invasion, multiplicity, and IHC staining for ER, PgR, and HER2. ER and PgR positivity were defined using Allred scores ranging from 3 to 8 based on IHC using antibodies to the ER (Immunotech, Marseille, France) and PgR (Novocastra Laboratories Ltd., Newcastle upon Tyne, UK). HER2 status was evaluated using a specific antibody (Dako, Glostrop, Denmark) and/or fluorescence *in situ* hybridization (FISH). Grades 0 and 1 for HER2, as assessed by IHC, were defined as a negative result, and grade 3 was defined as a positive result. Amplification of HER2 was confirmed by FISH if HER2 was rated as 2+ by IHC. HER2+ was defined as HER2-positive status irrespective of hormonal receptor status. TN breast cancer was defined as lack of expression of ER, PgR, and HER2.

### RNA extraction

All available hematoxylin and eosin (H & E)-stained sections from archival FFPE tissues were reviewed by two pathologists. Areas containing representative invasive breast carcinoma were outlined on the slide. Total RNA was extracted from 2 to 4 sections of 4-μm FFPE sections. With guidance from H & E-stained slides, non-tumor elements were removed by manual microdissection before transfer of tumor tissue to the extraction tube. Total RNA was extracted using the High Pure RNA Paraffin kit (Roche Diagnostic, Mannheim, Germany). RNA yield and purity were assessed using the NanoDrop ND-1000 Spectrophotometer (NanoDrop Technologies, Rockland, DE, USA). One sample with total RNA concentration less than 50 ng/L even after concentration using a SpeedVacTM concentrator (Thermo Scientific, Waltham, MA, USA) was excluded from downstream analysis because 200 ng of input RNA in a 5 uL volume was required for hybridization with 20 uL of the probe set mastermix.

### NanoString^®^ nCounter Assay

Gene expression was measured on the NanoString nCounter Analysis System (NanoString Technologies, Seattle, WA, USA). The system measures the relative abundance of each mRNA transcript of interest using a multiplexed hybridization assay and digital readouts of fluorescent barcoded probes that are hybridized to each transcript. An nCounter CodeSet (NanoString Technologies) containing a biotinylated capture probe for 252 target genes and five reference genes (Supplementary Table 2) and reporter probes attached to color barcode tags according to the nCounter^TM^ code-set design was hybridized in solution to 200 ng of total RNA for 18 h at 65 °C according to the manufacturer’s instructions.

Hybridized samples were loaded into the nCounter Prep Station for posthybridization processing. On the deck of the Prep Station, hybridized samples were purified and immobilized in a sample cartridge for data collection followed by quantification of target mRNA in each sample using the nCounter^TM^ Digital Analyzer. Quantified expression data were analyzed using NanoString nSolver Analysis Software v2.0. After performing image quality control using a predefined cutoff value, we excluded the outlier samples using a normalization factor based on the sum of positive control counts greater than 3-fold. The counts of the probes were then normalized using the geometric mean of five reference genes and log_2_ transformed for further analysis.

### Bioinformatics and Statistical Analysis for nCounter assay

For gene expression data from the NanoString nCounter assay, filtering of samples using quality control (QC) criteria was performed according to the manufacturer’s recommendations. Raw counts of QC-passed samples were normalized using five reference genes as internal controls (*GUSB*, *PUM1*, *TBP*, *TFRC*, and *TUBB*). Data were log_2_-transformed and used for further analysis. Student’s t-test was used to compare normalized expression values between groups classified according to clinical outcome. A chi-square test was used to compare categorical variables. *P* values were adjusted using the FDR method for multiple comparisons^54^. FDRs less than 0.05 were considered significantly different. We conducted PAC analysis to determine how well the expression pattern of genes discriminated between primary BC and BCBM. PAC analysis is a supervised method of identifying a subset of genes in a pathway or a gene set to optimize discriminative power for the phenotype^13^.

Intrinsic subtype classification was performed using the PAM50 predictor as described in Parker *et al*.^55^. To obtain more consistent results, we merged microarray expression data of TCGA breast cancers with our NanoString data after adjusting for batch effects using ComBat algorithm^56^, and applied the nearest PAM50 centroid algorithm Bioclassifier to predict PAM50 subtypes^55^. All statistical tests, plots, and PAM50 subtype prediction were conducted using R version 3.0.2 (http://www.R-project.org/).

### Remark guidelines

In reporting our study, we have adhered to the guidelines of an important methodological paper from 2005 entitled “Reporting recommendations for tumor marker prognostic studies (REMARK guidelines)”^57^,^58^.

## Additional Information

**How to cite this article**: Lee, J. Y. *et al*. Gene Expression Profiling of Breast Cancer Brain Metastasis. *Sci. Rep.* **6**, 28623; doi: 10.1038/srep28623 (2016).

## Supplementary Material

Supplementary Information

## Figures and Tables

**Table 1:** Baseline characteristics (n = 44).

	No.	%
Median age (range), years		
At initial diagnosis of BC	45 (22–64)	
At initial diagnosis of BCBM	48 (34–65)	
Menopausal status (n = 39)		
Premenopausal	31	79.5
Postmenopausal	8	20.5
Histology (n = 42)		
Invasive ductal carcinoma	37	88.1
Invasive lobular carcinoma	1	2.4
Others	4	9.5
Grade (n = 32)		
Low	0	0
Intermediate	11	34.4
High	21	65.6
T stage at initial diagnosis (n = 34)		
T1	13	38.2
T2	18	52.9
T3	3	8.8
T4	0	0
N stage at initial diagnosis (n = 35)		
N0	11	31.4
N1	12	34.3
N2	6	17.1
N3	6	17.1
Stage at initial diagnosis (n = 42)		
I	7	16.7
II	17	40.5
III	13	31.0
IV	5	11.9
Tumor subtype at initial diagnosis (n = 34)		
HR+	9	26.5
HER2+*	11	32.3
TNBC	14	41.2
Distant metastasis prior to the formation of brain lesions		
Yes	19	43.2
Site of metastasis Lung	8	42.1
Bone	7	36.8
Liver	6	31.6
Pleura	2	10.5
Adrenal gland	1	5.3
No	25	56.8

BC, breast cancer; BCBM, breast cancer brain metastasis; HR, hormone receptor (ER and/or PgR); TNBC, triple negative breast cancer.

*HER2-positive irrespective of HR status.

**Table 2:** Molecular subtype conversion of breast cancer in patient-matched pair samples (n = 17).

Pair No.	PAM50		IHC	
	Breast	Brain	Breast	Brain
1	LumA	LumB	ER+	TN
2	LumA	LumA	ER+	ER+
3	Basal	Basal	TN	TN
4	Her2	LumA	HER2+	HER2+
5	LumA	Her2	ER+	ER+
6	Her2	Her2	TN	TN
7	LumA	Her2	ER+	TN
8	Basal	Basal	TN	TN
9	Her2	Her2	HER2+	HER2+
10	Basal	Basal	TN	TN
11	LumA	LumB	ER+	ER+
12	Her2	Normal	HER2+	HER2+
13	Basal	Basal	TN	TN
14	Basal	Basal	TN	TN
15	LumA	Her2	HER2+	HER2+
16	Basal	Basal	TN	TN
18	Normal	Basal	TN	TN

IHC, immunohistochemistry, Lum, luminal; ER, estrogen receptor; TN, triple negative.
