# Supplementary material for: Gene Expression Profiling of Breast Cancer Brain Metastasis
Source: Sci Rep. 2016 Jun 24;6:28623. doi: 10.1038/srep28623 (PMC4919653; doi:10.1038/srep28623)

**Gene Expression Profiling of Breast Cancer Brain Metastasis**

Ji Yun Lee^1 *^, Kyunghee Park^2*^, Eunjin Lee^2^, TaeJin Ahn^2^, Hae Hyun Jung^3^, Sung Hee Lim^1^, Mineui Hong^4^, In-Gu Do^4^, Eun Yoon Cho^4^, Duk-Hwan Kim^5^, Ji-Yeon Kim^1^, Jin Seok Ahn^1^, Young-Hyuck Im^1,2^ and Yeon Hee Park^1,2^

^1^Division of Hematology-Oncology, Department of Medicine, Samsung Medical Center, Sungkyunkwan University School of Medicine, Seoul, Korea

^2^Samsung Genomic Institute, Samsung Biological Research Institute, Samsung Medical Center, Sungkyunkwan University School of Medicine, Seoul, Korea

^3^Biomedical Research Institute Samsung Biological Research Institute, Samsung Medical Center, Sungkyunkwan University School of Medicine, Seoul, Korea

^4^Center of Companion Diagnostics, Innovative Cancer Medicine Institute, Samsung Medical Center, Sungkyunkwan University School of Medicine, Seoul, Korea

^5^Department of Molecular Cell Biology, Samsung Biomedical Research Institute, Sungkyunkwan University School of Medicine, Suwon, Korea

*These authors contributed equally to this study.

**Table S1.** Molecular subtypes (n = 61)

| **Sample** | **Group** | **IHC** | **PAM50** |
| --- | --- | --- | --- |
| BB_001 | Breast | ER+ | LumA |
| BB_002 | Breast | ER+ | LumA |
| BB_003 | Breast | TN | Basal |
| BB_004 | Breast | HER2+ | Her2 |
| BB_005 | Breast | ER+ | LumA |
| BB_006 | Breast | TN | Her2 |
| BB_007 | Breast | ER+ | LumA |
| BB_008 | Breast | TN | Basal |
| BB_009 | Breast | HER2+ | Her2 |
| BB_010 | Breast | TN | Basal |
| BB_011 | Breast | ER+ | LumA |
| BB_012 | Breast | HER2+ | Her2 |
| BB_013 | Breast | TN | Basal |
| BB_014 | Breast | TN | Basal |
| BB_015 | Breast | HER2+ | LumA |
| BB_016 | Breast | TN | Basal |
| BB_017 | Breast | HER2+ | Her2 |
| BB_018 | Breast | TN | Normal |
| BB_019 | Breast | HER2+ | LumA |
| BB_020 | Breast | HER2+ | Her2 |
| BB_021 | Brain | TN | LumB |
| BB_022 | Brain | ER+ | LumA |
| BB_023 | Brain | TN | Basal |
| BB_024 | Brain | HER2+ | LumA |
| BB_025 | Brain | ER+ | Her2 |
| BB_026 | Brain | TN | Her2 |
| BB_027 | Brain | TN | Her2 |
| BB_028 | Brain | TN | Basal |
| BB_029 | Brain | HER2+ | Her2 |
| BB_030 | Brain | TN | Basal |
| BB_031 | Brain | ER+ | LumB |
| BB_032 | Brain | HER2+ | Normal |
| BB_033 | Brain | TN | Basal |
| BB_034 | Brain | ER+ | Basal |
| BB_035 | Brain | HER2+ | Her2 |
| BB_036 | Brain | TN | Basal |
| BB_038 | Brain | TN | Basal |
| BB_041 | Brain | TN | Basal |
| BB_042 | Brain | ER+ | LumB |
| BB_043 | Brain | TN | Her2 |
| BB_045 | Brain | HER2+ | Her2 |
| BB_046 | Brain | ER+ | LumA |
| BB_047 | Brain | TN | Basal |
| BB_048 | Brain | TN | Basal |
| BB_049 | Brain | HER2+ | Her2 |
| BB_050 | Brain | HER2+ | Her2 |
| BB_051 | Brain | ER+ | Basal |
| BB_052 | Brain | HER2+ | Her2 |
| BB_053 | Brain | HER2+ | Her2 |
| BB_054 | Brain | ER+ | Basal |
| BB_055 | Brain | ER+ | LumB |
| BB_056 | Brain | TN | Basal |
| BB_057 | Brain | TN | Basal |
| BB_058 | Brain | HER2+ | LumB |
| BB_059 | Brain | HER2+ | Her2 |
| BB_060 | Brain | TN | Basal |
| BB_061 | Brain | ER+ | LumA |
| BB_062 | Brain | HER2+ | Her2 |
| BB_063 | Brain | ER+ | LumB |
| BB_064 | Brain | ER+ | LumB |
| BB_065 | Brain | HER2+ | LumB |

**Table S2.** 257 nCounter gene list including PAM50 genes and 5 reference genes.

| **No.** | **Gene** | **Description** |
| --- | --- | --- |
| 1 | ABCB1 | ATP-binding cassette, sub-family B (MDR/TAP), member 1 |
| 2 | ABCC1 | ATP-binding cassette, sub-family C (CFTR/MRP), member 1 |
| 3 | ABCC2 | ATP-binding cassette, sub-family C (CFTR/MRP), member 2 |
| 4 | ABL1 | c-abl oncogene 1, non-receptor tyrosine kinase |
| 5 | ACTR3B | ARP3 actin-related protein 3 homolog B (yeast) |
| 6 | AKT1 | v-akt murine thymoma viral oncogene homolog 1 |
| 7 | AKT2 | v-akt murine thymoma viral oncogene homolog 2 |
| 8 | AKT3 | v-akt murine thymoma viral oncogene homolog 3 (protein kinase B, gamma) |
| 9 | ALCAM | activated leukocyte cell adhesion molecule |
| 10 | ALK | anaplastic lymphoma receptor tyrosine kinase |
| 11 | ANLN | anillin, actin binding protein |
| 12 | APC | adenomatous polyposis coli |
| 13 | APEX1 | APEX nuclease (multifunctional DNA repair enzyme) 1 |
| 14 | APOD | apolipoprotein D |
| 15 | AR | androgen receptor |
| 16 | ARID1A | AT rich interactive domain 1A (SWI-like) |
| 17 | ARID1B | AT rich interactive domain 1B (SWI1-like) |
| 18 | ARID2 | AT rich interactive domain 2 (ARID, RFX-like) |
| 19 | ATM | ataxia telangiectasia mutated |
| 20 | ATRX | alpha thalassemia/mental retardation syndrome X-linked |
| 21 | AURKA | aurora kinase A |
| 22 | AURKB | aurora kinase B |
| 23 | BAG1 | BCL2-associated athanogene |
| 24 | BCL2 | B-cell CLL/lymphoma 2 |
| 25 | BIRC5 | baculoviral IAP repeat containing 5 |
| 26 | BLVRA | biliverdin reductase A |
| 27 | BMP1 | bone morphogenetic protein 1 |
| 28 | BMP2 | bone morphogenetic protein 2 |
| 29 | BMX | BMX non-receptor tyrosine kinase |
| 30 | BRAF | v-raf murine sarcoma viral oncogene homolog B1 |
| 31 | BRCA1 | breast cancer 1, early onset |
| 32 | BRCA2 | breast cancer 2, early onset |
| 33 | CAV1 | caveolin 1, caveolae protein, 22kDa |
| 34 | CAV2 | caveolin 2 |
| 35 | CCNA1 | cyclin A1 |
| 36 | CCNA2 | cyclin A2 |
| 37 | CCNB1 | cyclin B1 |
| 38 | CCNB2 | cyclin B2 |
| 39 | CCND1 | cyclin D1 |
| 40 | CCND2 | cyclin D2 |
| 41 | CCNE1 | cyclin E1 |
| 42 | CD24 | CD24 molecule |
| 43 | CD44 | CD44 molecule (Indian blood group) |
| 44 | CD68 | CD68 molecule |
| 45 | CDC20 | cell division cycle 20 homolog (S. cerevisiae) |
| 46 | CDC6 | cell division cycle 6 homolog (S. cerevisiae) |
| 47 | CDCA1 | NUF2, NDC80 kinetochore complex component, homolog (S. cerevisiae) |
| 48 | CDH1 | cadherin 1, type 1, E-cadherin (epithelial) |
| 49 | CDH2 | cadherin 2, type 1, N-cadherin (neuronal) |
| 50 | CDH3 | cadherin 3, type 1, P-cadherin (placental) |
| 51 | CDK4 | cyclin-dependent kinase 4 |
| 52 | CDK6 | cyclin-dependent kinase 6 |
| 53 | CDKN1A | cyclin-dependent kinase inhibitor 1A (p21, Cip1) |
| 54 | CDKN1B | Cyclin-dependent kinase inhibitor 1B (p27, Kip1) |
| 55 | CDKN2A | cyclin-dependent kinase inhibitor 2A (melanoma, p16, inhibits CDK4) |
| 56 | CENPF | centromere protein F, 350/400ka (mitosin) |
| 57 | CEP55 | centrosomal protein 55kDa |
| 58 | CLDN3 | claudin 3 |
| 59 | CLDN4 | claudin 4 |
| 60 | CLDN7 | claudin 7 |
| 61 | CLTC | clathrin, heavy chain (Hc) |
| 62 | CSF1R | colony stimulating factor 1 receptor |
| 63 | CTNNB1 | catenin (cadherin-associated protein), beta 1, 88kDa |
| 64 | CTSL2 | cathepsin L2 |
| 65 | CXCL12 | chemokine (C-X-C motif) ligand 12 (stromal cell-derived factor 1) |
| 66 | CXCR2 | interleukin 8 receptor, beta |
| 67 | CXCR4 | chemokine (C-X-C motif) receptor 4 |
| 68 | CXXC5 | CXXC finger 5 |
| 69 | DDR2 | discoidin domain receptor tyrosine kinase 2 |
| 70 | DHCR24 | 24-dehydrocholesterol reductase |
| 71 | DUSP4 | dual specificity phosphatase 4 |
| 72 | EGFR | epidermal growth factor receptor |
| 73 | EHF | ets homologous factor |
| 74 | ELF1 | E74-like factor 1 (ets domain transcription factor) |
| 75 | ELF2 | E74-like factor 2 (ets domain transcription factor) |
| 76 | ELF3 | E74-like factor 3 (ets domain transcription factor, epithelial-specific ) |
| 77 | ELF4 | E74-like factor 4 (ets domain transcription factor) |
| 78 | ELF5 | E74-like factor 5 (ets domain transcription factor) |
| 79 | ELK1 | ELK1, member of ETS oncogene family |
| 80 | ELK3 | ELK3, ETS-domain protein (SRF accessory protein 2) |
| 81 | ELK4 | ELK4, ETS-domain protein (SRF accessory protein 1) |
| 82 | EPAS1 | endothelial PAS domain protein 1 |
| 83 | EPHA2 | EPH receptor A2 |
| 84 | EPHB4 | EPH receptor B4 |
| 85 | ERBB2 | v-erb-b2 erythroblastic leukemia viral oncogene homolog 2, neuro/glioblastoma derived oncogene homolog (avian) |
| 86 | ERBB3 | v-erb-b2 erythroblastic leukemia viral oncogene homolog 3 (avian) |
| 87 | ERBB4 | v-erb-a erythroblastic leukemia viral oncogene homolog 4 (avian) |
| 88 | ERF | Ets2 repressor factor |
| 89 | ERG | v-ets erythroblastosis virus E26 oncogene homolog (avian) |
| 90 | ESR1 | estrogen receptor 1 |
| 91 | ETS1 | v-ets erythroblastosis virus E26 oncogene homolog 1 (avian) |
| 92 | ETS2 | v-ets erythroblastosis virus E26 oncogene homolog 2 (avian) |
| 93 | ETV1 | ets variant 1 |
| 94 | ETV2 | ets variant 2 |
| 95 | ETV3 | ets variant 3 |
| 96 | ETV4 | ets variant 4 |
| 97 | ETV5 | ets variant 5 |
| 98 | ETV6 | ets variant 6 |
| 99 | ETV7 | ets variant 7 |
| 100 | EXO1 | exonuclease 1 |
| 101 | EZH2 | enhancer of zeste homolog 2 (Drosophila) |
| 102 | FASN | fatty acid synthase |
| 103 | FBXW7 | F-box and WD repeat domain containing 7 |
| 104 | FEV | FEV (ETS oncogene family) |
| 105 | FGFR1 | fibroblast growth factor receptor 1 |
| 106 | FGFR2 | fibroblast growth factor receptor 2 |
| 107 | FGFR3 | fibroblast growth factor receptor 3 |
| 108 | FGFR4 | fibroblast growth factor receptor 4 |
| 109 | FLI1 | Friend leukemia virus integration 1 |
| 110 | FLT1 | fms-related tyrosine kinase 1 (vascular endothelial growth factor/vascular permeability factor receptor) |
| 111 | FLT3 | fms-related tyrosine kinase 3 |
| 112 | FLT4 | fms-related tyrosine kinase 4 |
| 113 | FOS | FBJ murine osteosarcoma viral oncogene homolog |
| 114 | FOXA1 | forkhead box A1 |
| 115 | FOXC1 | forkhead box C1 |
| 116 | FZD7 | frizzled homolog 7 (Drosophila) |
| 117 | GABPA | GA binding protein transcription factor, alpha subunit 60kDa |
| 118 | GAPDH | glyceraldehyde-3-phosphate dehydrogenase |
| 119 | GNA11 | guanine nucleotide binding protein (G protein), alpha 11 (Gq class) |
| 120 | GNAQ | guanine nucleotide binding protein (G protein), q polypeptide |
| 121 | GNAS | GNAS complex locus |
| 122 | GPR160 | G protein-coupled receptor 160 |
| 123 | GRB7 | growth factor receptor-bound protein 7 |
| 124 | GSTM1 | glutathione S-transferase M1 |
| 125 | GUSB | glucuronidase, beta |
| 126 | HIF1A | hypoxia inducible factor 1, alpha subunit (basic helix-loop-helix transcription factor) |
| 127 | HNF1A | HNF1 homeobox A |
| 128 | HRAS | v-Ha-ras Harvey rat sarcoma viral oncogene homolog |
| 129 | HSP90AA1 | heat shock protein 90kDa alpha (cytosolic), class A member 1 |
| 130 | HSPA1A | heat shock 70kDa protein 1A |
| 131 | HSPB1 | heat shock 27kDa protein 1 |
| 132 | ID1 | inhibitor of DNA binding 1, dominant negative helix-loop-helix protein |
| 133 | ID2 | inhibitor of DNA binding 2, dominant negative helix-loop-helix protein |
| 134 | ID4 | inhibitor of DNA binding 4, dominant negative helix-loop-helix protein |
| 135 | IDH1 | isocitrate dehydrogenase 1 (NADP+), soluble |
| 136 | IDH2 | isocitrate dehydrogenase 2 (NADP+), mitochondrial |
| 137 | IGF1R | insulin-like growth factor 1 receptor |
| 138 | INPP4B | inositol polyphosphate-4-phosphatase, type II, 105kDa |
| 139 | ITK | IL2-inducible T-cell kinase |
| 140 | JAG1 | jagged 1 (Alagille syndrome) |
| 141 | JAK1 | Janus kinase 1 (a protein tyrosine kinase) |
| 142 | JAK2 | Janus kinase 2 (a protein tyrosine kinase) |
| 143 | JAK3 | Janus kinase 3 (a protein tyrosine kinase, leukocyte) |
| 144 | JUN | jun oncogene |
| 145 | KDR | kinase insert domain receptor (a type III receptor tyrosine kinase) |
| 146 | KIF2C | kinesin family member 2C |
| 147 | KIT | v-kit Hardy-Zuckerman 4 feline sarcoma viral oncogene homolog |
| 148 | KNTC2 | NDC80 homolog, kinetochore complex component (S. cerevisiae) |
| 149 | KRAS | v-Ki-ras2 Kirsten rat sarcoma viral oncogene homolog |
| 150 | KRT14 | keratin 14 |
| 151 | KRT17 | keratin 17 |
| 152 | KRT5 | keratin 5 |
| 153 | KRT6A | keratin 6A |
| 154 | MAP2K1 | mitogen-activated protein kinase kinase 1 |
| 155 | MAPK1 | mitogen-activated protein kinase 1 |
| 156 | MAPK14 | mitogen-activated protein kinase 14 |
| 157 | MAPK3 | mitogen-activated protein kinase 3 |
| 158 | MAPK8 | mitogen-activated protein kinase 8 |
| 159 | MAPT | microtubule-associated protein tau |
| 160 | MCL1 | myeloid cell leukemia sequence 1 (BCL2-related) |
| 161 | MDM2 | Mdm2 p53 binding protein homolog (mouse) |
| 162 | MDM4 | Mdm4 p53 binding protein homolog (mouse) |
| 163 | MELK | maternal embryonic leucine zipper kinase |
| 164 | MET | met proto-oncogene (hepatocyte growth factor receptor) |
| 165 | MIA | melanoma inhibitory activity |
| 166 | MKI67 | antigen identified by monoclonal antibody Ki-67 |
| 167 | MLH1 | mutL homolog 1, colon cancer, nonpolyposis type 2 (E. coli) |
| 168 | MLPH | melanophilin |
| 169 | MME | membrane metallo-endopeptidase |
| 170 | MMP10 | matrix metallopeptidase 10 (stromelysin 2) |
| 171 | MMP11 | matrix metallopeptidase 11 (stromelysin 3) |
| 172 | MMP13 | matrix metallopeptidase 13 (collagenase 3) |
| 173 | MMP2 | matrix metallopeptidase 2 (gelatinase A, 72kDa gelatinase, 72kDa type IV collagenase) |
| 174 | MMP9 | matrix metallopeptidase 9 (gelatinase B, 92kDa gelatinase, 92kDa type IV collagenase) |
| 175 | MPL | myeloproliferative leukemia virus oncogene |
| 176 | MTOR | mechanistic target of rapamycin (serine/threonine kinase) |
| 177 | MYBL2 | v-myb myeloblastosis viral oncogene homolog (avian)-like 2 |
| 178 | MYC | v-myc myelocytomatosis viral oncogene homolog (avian) |
| 179 | NAT1 | N-acetyltransferase 1 (arylamine N-acetyltransferase) |
| 180 | NF1 | neurofibromin 1 |
| 181 | NFKB1 | nuclear factor of kappa light polypeptide gene enhancer in B-cells 1 |
| 182 | NFKB2 | nuclear factor of kappa light polypeptide gene enhancer in B-cells 2 (p49/p100) |
| 183 | NOTCH1 | notch 1 |
| 184 | NOTCH2 | notch 2 |
| 185 | NOTCH3 | notch 3 |
| 186 | NOTCH4 | notch 4 |
| 187 | NPM1 | nucleophosmin (nucleolar phosphoprotein B23, numatrin) |
| 188 | NRAS | neuroblastoma RAS viral (v-ras) oncogene homolog |
| 189 | NTRK1 | neurotrophic tyrosine kinase, receptor, type 1 |
| 190 | OLIG2 | oligodendrocyte lineage transcription factor 2 |
| 191 | ORC6L | origin recognition complex, subunit 6 like (yeast |
| 192 | PAK1 | p21 protein (Cdc42/Rac)-activated kinase 1 |
| 193 | PCNA | proliferating cell nuclear antigen |
| 194 | PDGFRA | platelet-derived growth factor receptor, alpha polypeptide |
| 195 | PDGFRB | platelet-derived growth factor receptor, beta polypeptide |
| 196 | PGR | progesterone receptor |
| 197 | PHGDH | phosphoglycerate dehydrogenase |
| 198 | PIK3CA | phosphoinositide-3-kinase, catalytic, alpha polypeptide |
| 199 | PIK3R1 | [phosphoinositide-3-kinase, regulatory subunit 1 (alpha)](javascript:if(window.name=='') { window.location.href='./nil'; } else { doaction(null, 94877, 1); }) |
| 200 | POU5F1 | POU class 5 homeobox 1 |
| 201 | PTCH1 | patched homolog 1 (Drosophila) |
| 202 | PTCH2 | patched homolog 2 (Drosophila) |
| 203 | PTEN | phosphatase and tensin homolog |
| 204 | PTK2 | PTK2 protein tyrosine kinase 2 |
| 205 | PTPN11 | protein tyrosine phosphatase, non-receptor type 11 |
| 206 | PTTG1 | pituitary tumor-transforming 1 |
| 207 | PUM1 | pumilio homolog 1 (Drosophila) |
| 208 | RB1 | retinoblastoma 1 |
| 209 | REL | v-rel reticuloendotheliosis viral oncogene homolog (avian) |
| 210 | RELB | v-rel reticuloendotheliosis viral oncogene homolog B |
| 211 | RET | ret proto-oncogene |
| 212 | ROS1 | c-ros oncogene 1 , receptor tyrosine kinase |
| 213 | RRM2 | ribonucleotide reductase M2 polypeptide |
| 214 | SCUBE2 | gnal peptide, CUB domain, EGF-like 2 |
| 215 | SFRP1 | secreted frizzled-related protein 1 |
| 216 | SLC39A6 | solute carrier family 39 (zinc transporter), member 6 |
| 217 | SMAD2 | SMAD family member 2 |
| 218 | SMAD4 | SMAD family member 4 |
| 219 | SMARCB1 | SWI/SNF related, matrix associated, actin dependent regulator of chromatin, subfamily b, member 1 |
| 220 | SMO | smoothened homolog (Drosophila) |
| 221 | SNAI1 | snail homolog 1 (Drosophila) |
| 222 | SNAI2 | snail homolog 2 (Drosophila) |
| 223 | SOX2 | SRY (sex determining region Y)-box 2 |
| 224 | SPDEF | SAM pointed domain containing ets transcription factor |
| 225 | SPI1 | spleen focus forming virus (SFFV) proviral integration oncogene spi1 |
| 226 | SPIB | Spi-B transcription factor (Spi-1/PU.1 related) |
| 227 | SPIC | Spi-C transcription factor (Spi-1/PU.1 related) |
| 228 | SRC | v-src sarcoma (Schmidt-Ruppin A-2) viral oncogene homolog (avian) |
| 229 | STAT3 | signal transducer and activator of transcription 3 (acute-phase response factor) |
| 230 | STK11 | serine/threonine kinase 11 |
| 231 | SYK | spleen tyrosine kinase |
| 232 | TBP | TATA box binding protein |
| 233 | TEK | TEK tyrosine kinase, endothelial |
| 234 | TFRC | transferrin receptor (p90, CD71) |
| 235 | TIE1 | tyrosine kinase with immunoglobulin-like and EGF-like domains 1 |
| 236 | TIMP2 | TIMP metallopeptidase inhibitor 2 |
| 237 | TIMP3 | TIMP metallopeptidase inhibitor 3 |
| 238 | TIMP4 | TIMP metallopeptidase inhibitor 4 |
| 239 | TMEM45B | transmembrane protein 45B |
| 240 | TOP1 | topoisomerase (DNA) I |
| 241 | TP53 | tumor protein p53 |
| 242 | TP63 | tumor protein p63 |
| 243 | TUBB | tubulin, beta |
| 244 | TWIST1 | twist homolog 1 (Drosophila) |
| 245 | TYMS | thymidylate synthetase |
| 246 | UBE2C | ubiquitin-conjugating enzyme E2C |
| 247 | UBE2T | ubiquitin-conjugating enzyme E2T (putative) |
| 248 | VCAM1 | vascular cell adhesion molecule 1 |
| 249 | VEGFA | vascular endothelial growth factor A |
| 250 | VHL | von Hippel-Lindau tumor suppressor |
| 251 | VIM | vimentin |
| 252 | WNT3A | wingless-type MMTV integration site family, member 3A |
| 253 | WNT5A | wingless-type MMTV integration site family, member 5A |
| 254 | WNT5B | wingless-type MMTV integration site family, member 5B |
| 255 | YBX1 | Y box binding protein 1 |
| 256 | ZEB1 | zinc finger E-box binding homeobox 1 |
| 257 | ZEB2 | zinc finger E-box binding homeobox 2 |

Green bar, PAM50 gene

**Table S3.** Genes differentially expressed between primary breast cancer and breast cancer brain metastasis (n = 61).

| **Gene** | **Brain** | **Breast** | **P value** | **FDR** | **diff** |
| --- | --- | --- | --- | --- | --- |
| **Upregulated in primary BC** | | | | | |
| MME | 3.4 | 7.073 | 3.09.E-12 | 4.16.E-10 | 3.673 |
| MMP2 | 7.194 | 10.692 | 3.08.E-12 | 4.16.E-10 | 3.497 |
| CXCL12 | 7.904 | 10.637 | 7.81.E-12 | 7.03.E-10 | 2.734 |
| PDGFRA | 6.514 | 9.043 | 1.72.E-10 | 1.09.E-08 | 2.529 |
| VCAM1 | 5.324 | 8.264 | 3.89.E-10 | 1.75.E-08 | 2.94 |
| MMP13 | 2.293 | 7.04 | 1.84.E-09 | 7.11.E-08 | 4.746 |
| ITK | 3.666 | 7.557 | 2.41.E-09 | 8.15.E-08 | 3.891 |
| MMP11 | 6.983 | 10.593 | 6.52.E-09 | 1.96.E-07 | 3.609 |
| MMP9 | 7.706 | 10.959 | 2.03.E-08 | 5.48.E-07 | 3.253 |
| SPIB | 2.683 | 6.678 | 1.44.E-06 | 2.28.E-05 | 3.995 |
| SCUBE2 | 4.745 | 8.388 | 3.82.E-06 | 5.16.E-05 | 3.643 |
| TP63 | 3.983 | 6.77 | 6.70.E-06 | 8.61.E-05 | 2.787 |
| FLT3 | 2.373 | 5.369 | 7.82.E-06 | 9.59.E-05 | 2.996 |
| JAK3 | 6.261 | 8.462 | 8.77.E-06 | 1.03.E-04 | 2.201 |
| KRT14 | 3.511 | 8.331 | 2.71.E-05 | 2.61.E-04 | 4.821 |
| KRT17 | 7.055 | 10.15 | 1.37.E-04 | 9.22.E-04 | 3.096 |
| KRT5 | 4.56 | 8.678 | 1.48.E-04 | 9.74.E-04 | 4.118 |
| SFRP1 | 5.825 | 9.038 | 5.42.E-04 | 3.11.E-03 | 3.213 |
| SPIC | 1.19 | 3.785 | 1.44.E-03 | 6.93.E-03 | 2.594 |
| PGR | 2.399 | 4.902 | 7.43.E-03 | 2.65.E-02 | 2.503 |
| **Downregulated in primary BC** | | | | | |
| OLIG2 | 4.315 | 0.641 | 2.02.E-10 | 1.09.E-08 | -3.674 |
| SOX2 | 5.701 | 1.66 | 4.34.E-08 | 9.75.E-07 | -4.041 |

**Table S4.** Molecular subtypes of group B.

| **Sample** | **Group** | **IHC** | **PAM50** |
| --- | --- | --- | --- |
| BB_023 | Brain | TN | Basal |
| BB_008 | Breast | TN | Basal |
| BB_028 | Brain | TN | Basal |
| BB_029 | Brain | HER2+ | Her2 |
| BB_010 | Breast | TN | Basal |
| BB_032 | Brain | HER2+ | Normal |
| BB_036 | Brain | TN | Basal |
| BB_047 | Brain | TN | Basal |
| BB_048 | Brain | TN | Basal |
| BB_051 | Brain | ER+ | Basal |
| BB_056 | Brain | TN | Basal |
| BB_057 | Brain | TN | Basal |

**Supplementary Fig. 1** Sample resources.


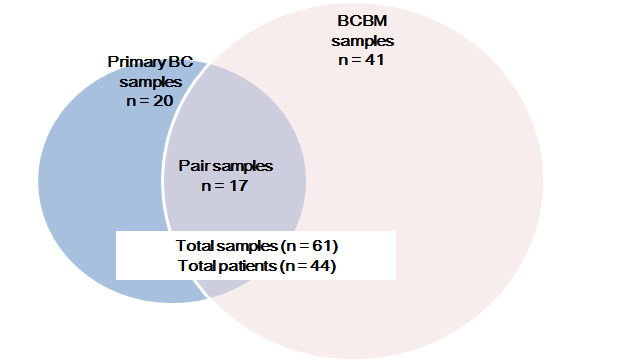


**Supplementary Fig. 2** Survival curves according to the subtype. (A) Overall survival from time of initial diagnosis of BC. (B) Overall survival from time of BCBM.

**
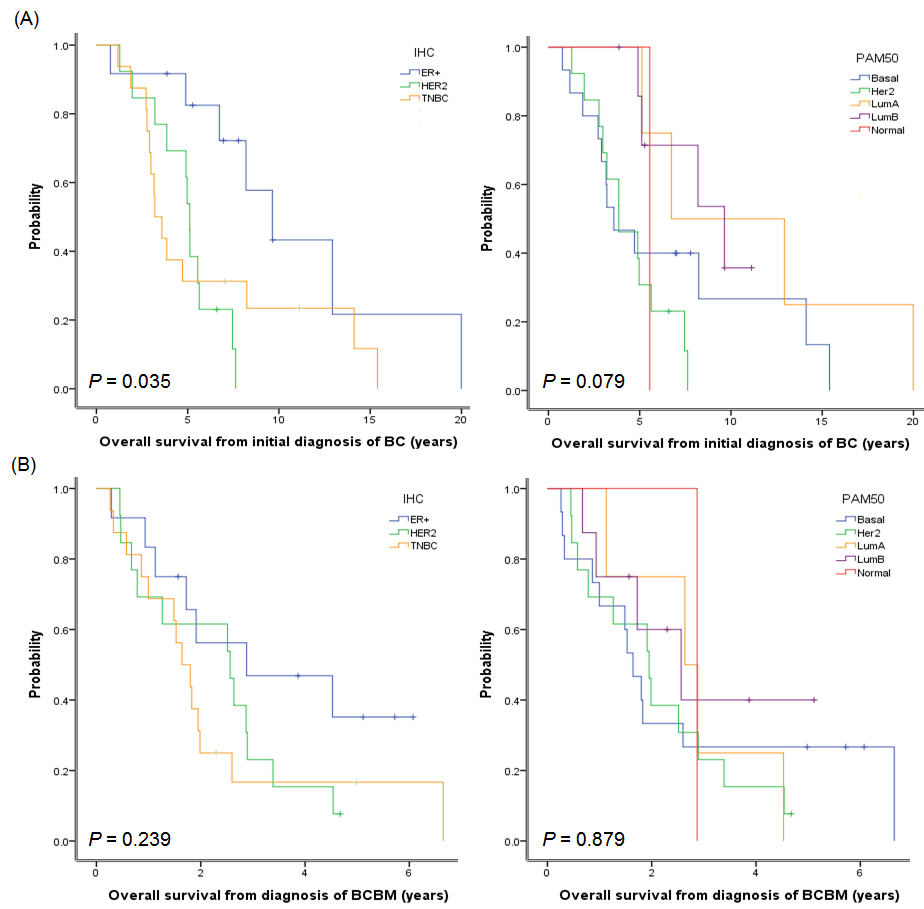
**

**Supplementary Fig. 3** Outline of bioinformatics algorithms for identification of 22 genes

**
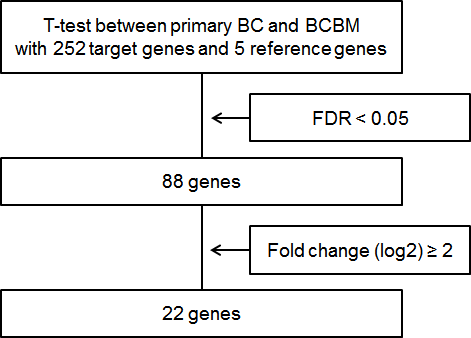
**

**Supplementary Fig. 4** Heat map showing differences in the expression patterns of 252 genes in patient-matched pair samples with absolute fold change ≥ 2 and *FDR* < 0.05. Comparisons were analyzed using Student’s t-test.

**
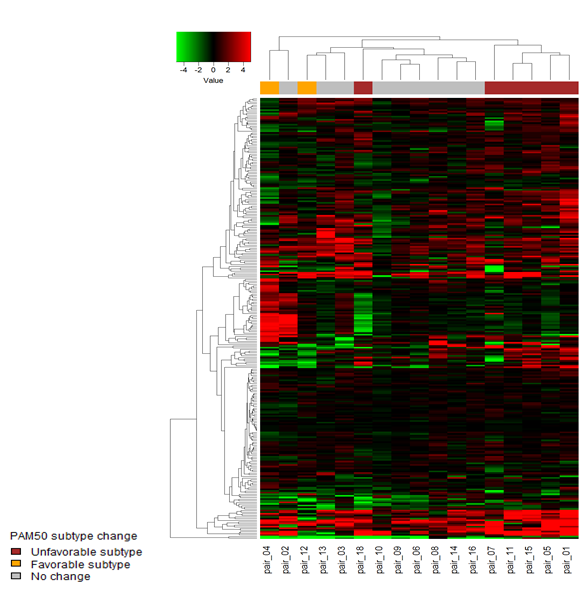
**

**Supplementary Fig. 5** Heat map showing differences in the expression patterns of PAM50 genes between mutant TP53 and wild-type with absolute fold change ≥ 2 and *FDR* < 0.05. Comparisons were analyzed using Student’s t-test.

**
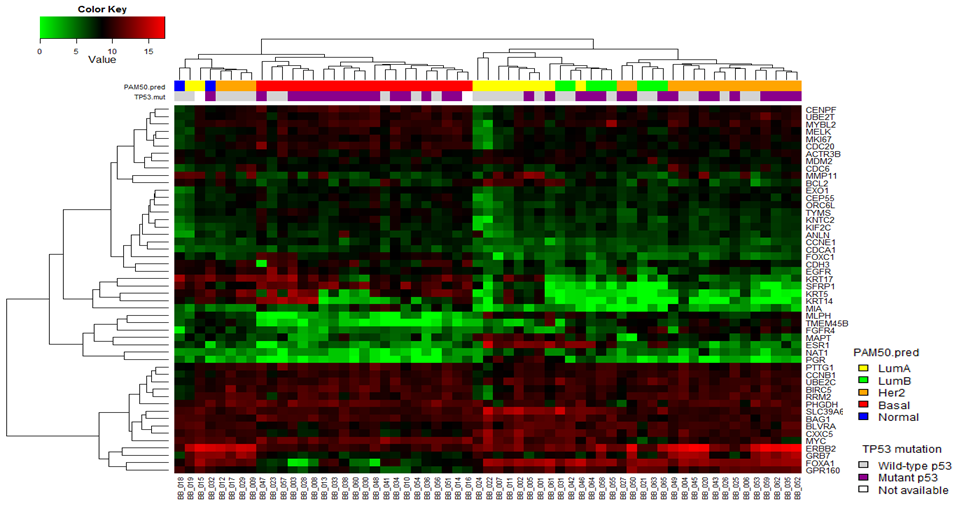
**

**Supplementary Fig. 6** Gene expression profiles related to TP53 mutation status. (A) Total cohort. (B) BCBM cohort. Gene expression profiles of the 25 mutant TP53 compared to those of the 16 wild-type TP53. Volcano plots show the distribution of the fold changes in gene expression. Genes with absolute fold change ≥ 2 and *P* value < 0.01 are indicated in red (high expression in patient with TP53 mutation) and blue (low expression in patient with TP53 mutation). Comparisons were analyzed using Student’s t-test.

**
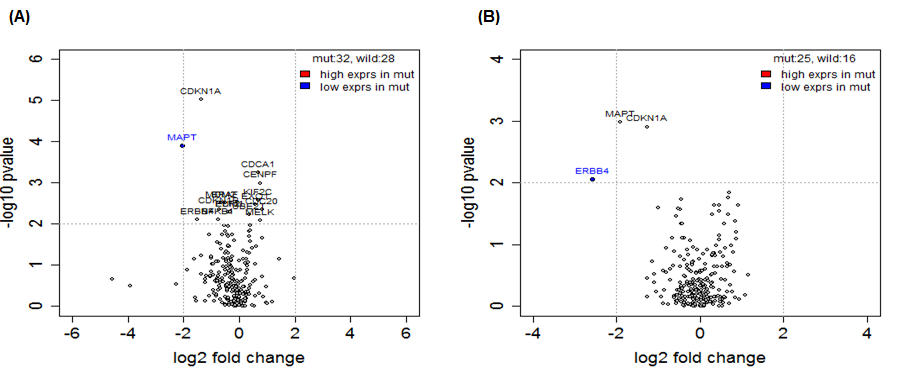
**

**Supplementary Fig. 7** The linear regression analysis between levels of CXCL12 and CXCR4 (correlation coefficient = 0.52). Dotted line means median gene expression levels of CXCL12 and CXCR4, respectively.


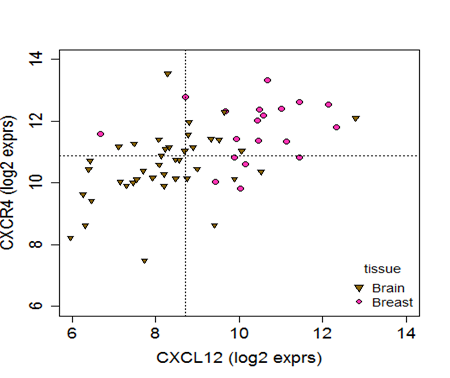

Supplement: Supplementary Information [file srep28623-s1.doc]
